# Understanding Image Representations by Measuring Their Equivariance and Equivalence

**DOI:** 10.1007/s11263-018-1098-y

**Published:** 2018-05-18

**Authors:** Karel Lenc, Andrea Vedaldi

**Affiliations:** Department of Engineering Science, University of Oxford, Oxford, UK

**Keywords:** Image representations, Geometric equivariance, Equivalent representations, Convolutional neural networks

## Abstract

Despite the importance of image representations such as histograms of oriented gradients and deep Convolutional Neural Networks (CNN), our theoretical understanding of them remains limited. Aimed at filling this gap, we investigate two key mathematical properties of representations: equivariance and equivalence. Equivariance studies how transformations of the input image are encoded by the representation, invariance being a special case where a transformation has no effect. Equivalence studies whether two representations, for example two different parameterizations of a CNN, two different layers, or two different CNN architectures, share the same visual information or not. A number of methods to establish these properties empirically are proposed, including introducing transformation and stitching layers in CNNs. These methods are then applied to popular representations to reveal insightful aspects of their structure, including clarifying at which layers in a CNN certain geometric invariances are achieved and how various CNN architectures differ. We identify several predictors of geometric and architectural compatibility, including the spatial resolution of the representation and the complexity and depth of the models. While the focus of the paper is theoretical, direct applications to structured-output regression are demonstrated too.

## Introduction

Image representations have been a key focus of the research in computer vision for at least two decades. Notable examples include textons (Leung and Malik 2001), histogram of oriented gradients (SIFT Lowe 2004) and HOG Dalal and Triggs 2005), bag of visual words (Csurka et al. 2004; Sivic and Zisserman 2003), sparse (Yang et al. 2010) and local coding (Wang et al. 2010), super vector coding (Zhou et al. 2010), VLAD (Jégou et al. 2010), Fisher Vectors (Perronnin and Dance 2006), and, more recently, modern deep neural networks (Krizhevsky et al. 2012; Sermanet et al. 2014; Zeiler and Fergus 2013). Despite this extensive research effort, the development of image representations remains largely empirical, and our theoretical understanding of them is still limited. It is generally believed that a good representation should combine invariance and discriminability, but this characterization is rather vague; furthermore, it is often unclear what invariances are captured by existing representations and how they are obtained.

**Fig. 1:**
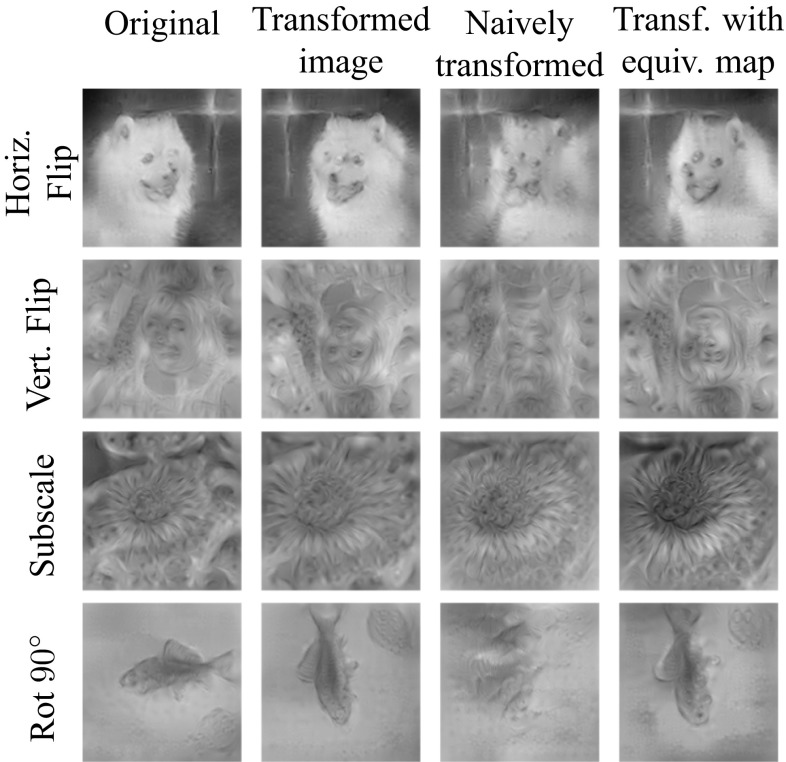
Equivariant transformation of CNN features. First column: features of a convolutional neural network (representation after four convolutional layers, C4) visualized with the method of Mahendran and Vedaldi (2016). Second column: Visualizing the features after transforming the image. Third column: Visualizing the features after the naive geometric transformation of the representation (spatial permutation only). Last column: Visualized transformed features using an equivariant transformation which additionally re-projects network feature channels learned using the method of Sect. 5

In this work, we formally investigate image representations in terms of their properties. In full generality, a *representation*$\phi$ is a function mapping an image $\mathbf {x}$ to a vector $\phi (\mathbf {x})\in \mathbb {R}^d$ and our goal is to *establish important statistical properties* of such functions. We focus on two such properties. The first one is **equivariance**, which looks at how the representation output *changes upon transformations of the input image*. We demonstrate that most representations, including HOG and most of the layers in deep neural networks, change in a *easily predictable* manner with geometric transformations of the input (Fig. 1). We show that such equivariant transformations can be learned empirically from data (Sect. 5.1) and that, importantly, they amount to simple linear transformations of the representation output (Sects. 5.2 and 5.3). In the case of convolutional networks, we obtain this by introducing and learning a new *transformation layer*. As a special case of equivariance, by analyzing the learned equivariant transformations we are also able to find and characterize the **invariances** of the representation. This allows us to quantify geometric invariance and to show how it builds up with the representation depth.

The second part of the manuscript investigates another property, **equivalence**, which looks at whether different representations, such as different neural networks, capture similar information or not. In the case of CNNs, in particular, the non-convex nature of learning means that the *same CNN architecture* may result in different models even when retrained on the *same data*. The question then is whether the resulting differences are substantial or just superficial. To answer this question, we propose to learn *stitching layers* that allow swapping parts of different architectures, rerouting information between them. Equivalence and coverage is then established if the resulting “Franken-CNNs” perform as well as the original ones (Sect. 6.2).

This paper extends the original conference paper (Lenc and Vedaldi 2015) substantially, by providing extensive results on recent deep neural network architectures, more analysis, and better visualizations. For equivariance, the paper investigates new formulations using alternative loss definitions as well as element-wise feature invariance. For equivalence, the paper systematically explores the equivalence between all layers of neural networks, analyzing for the first time the compatibility between different layers of different neural network architectures.

The rest of the paper is organized as follows. Section 3 discusses properties of selection of image representations. Section 5 discusses methods to learn empirically representation equivariance and invariance and presents experiments on shallow (Sect. 5.2) and deep (Sect. 5.3) representations. We also present a simple application of such results to structured-output regression in Sect. 5.4. In Sect. 6.2 we study the representation equivalence and show the relation between different deep image representations. Finally, Sect. 7 summarizes our findings.

## Related Work

The problem of designing invariant or equivariant features has been widely explored in computer vision, as it is a common task to remove nuisance factors from the data (both geometric and photometric).

Invariance to geometric nuisance factors is traditionally achieved with either pose normalization, or by folding an equivariant representation over a group (e.g. by averaging, max-pooling or by exploiting function symmetries) (Cohen and Welling 2016). Both of these principles are taken into account in the architecture of deep CNNs, including the design by Krizhevsky et al. (2012) and related state-of-the-art architectures (He et al. 2016; Simonyan et al. 2013), mainly for translation invariance which can be extended for different groups as well (Cohen and Welling 2016; Dieleman et al. 2015). This is even made more explicit in the *scattering transform* of Sifre and Mallat (2013). For pose normalization or feature folding the aim is to obtain invariant image features such that a non-invariant classifier can be used. However, in case of CNNs, the goal is to get an end-to-end *invariant classifier* and little is known of how and where these models achieve invariance to other nuisance factors present in the data (such as horizontal flipping).

There are many examples of the general pose normalization methodology in computer vision applications. One of the common approaches is to sample the nuisance feature space sparsely with various “detectors”—such as local feature detectors with different normalization schemes  (Lindeberg 1998; Lowe 1999; Mikolajczyk and Schmid 2003), bounding box proposals (Uijlings et al. 2013; Zitnick and Dollar 2014) or a direct regression of the normalized frame (Jaderberg et al. 2015; Ren et al. 2015). Another option is to sample the feature space densely using a grid search (Dalal and Triggs 2005; Felzenszwalb et al. 2009).^1^ It is always the detected geometric “frame” which is used to normalize either image or features in order to obtain invariant representations. However, in order to be able to normalize the features (due to computational constraints), the features need to be *equivariant* to the selected geometry factors. A number of authors have looked at incorporating equivariance explicitly in the representations (Schimdt and Roth 2012a; Sohn and Lee 2012).

A second approach to achieving invariance to a group of transformations is to fold the equivariant representation along the manifold induced by the nuisance transformation. This can be as simple as averaging the features (Anselmi et al. 2016), max-pooling (Laptev et al. 2016; Cohen and Welling 2016) or simply by exploiting the group symmetry [such as ignoring the gradient ‘sign’ in Dalal and Triggs (2005) for vertical flip invariance].

In all these examples, invariance is a design aim that may or may not be achieved by a given architecture. By contrast, our aim is *not* to propose yet another mechanism to learn invariances (Anselmi et al. 2016; Bruna and Mallat 2013; Huang et al. 2007) or equivariance (Dieleman et al. 2016; Schmidt and Roth 2012b), but rather a method to systematically tease out invariance, equivariance, and other properties that a given representation may have. To the best of our knowledge, there is very limited work in conducting this type of analysis. Perhaps the works most closely related to ours only study invariances of neural networks to specific image transformations (Goodfellow et al. 2009; Zeiler and Fergus 2013). In Aubry and Russell (2015), the authors train networks on computer generated imagery to visually investigate the manifold in the feature space induced by underlying object transformation (such as rotation, style *etc.*). They show that across layers, the invariance to viewpoint increases with depth (by studying invariances and intrinsic dimensionality), which corroborates our findings. However, differently to this work, we attempt to find whether there exists a transformation in feature space for the whole training dataset instead of quantitative statistics on its subset. We believe this work is the first to functionally characterize and quantify these properties in a systematic manner, as well as being the first to investigate the equivalence of different representations.

The equivariance maps and steerable filters (Freeman and Adelson 1991) share some of the underlying theory. While conceptually similar, this work searches for linear maps of existing representations, instead of designing representations to achieve steerability. In fact, some more recent works have attempted to design steerable CNN representations (Cohen and Welling 2017) for CIFAR10 dataset (Krizhevsky and Hinton 2009).

Another property of image representations studied in this work is equivalence and covering, which tackles the relationship between different representations. Yosinski et al. (2014), the authors study the transferability of CNN features between different tasks by retraining various parts of the networks. While this may seem similar to our equivalence study of networks trained for different tasks, we do not change existing representations or train new features, we only study the relationship between them.

The work Li et al. (2015), published one year after our original manuscript (Lenc and Vedaldi 2015), studies different ways how to find equivalence between networks trained with a different initialization with the goal of investigating the common factors of different networks quantitatively. While this work is similar to our equivalence chapter, our goal is to find relationship between representations of different layers and various deep CNN networks with architectural differences or trained for different tasks in order to understand better the geometry of the representations.

## Image Representations

An *image representation*$\phi$ associates to an image $\mathbf {x}$ a vector $\phi (\mathbf {x})\in \mathbb {R}^d$ that encodes the image content in a manner useful for tasks such as classification or regression. We distinguish two important families of representations: traditional “handcrafted” representations such as SIFT and HOG (Sect. 3.1) and modern learnable deep neural networks (Sect. 3.2).

### Traditional Image Representations

Before the advent of modern deep neural networks, computer vision researchers proposed various image representations such as textons (Leung and Malik 2001), histogram of oriented gradients (SIFT Lowe 2004 and HOG Dalal and Triggs 2005), bag of visual words (BoVW) (Csurka et al. 2004; Sivic and Zisserman 2003), sparse (Yang et al. 2010) and local coding (Wang et al. 2010), super vector coding (Zhou et al. 2010), VLAD (Jégou et al. 2010), Fisher Vectors (Perronnin and Dance 2006), and many others.

**Fig. 2:**
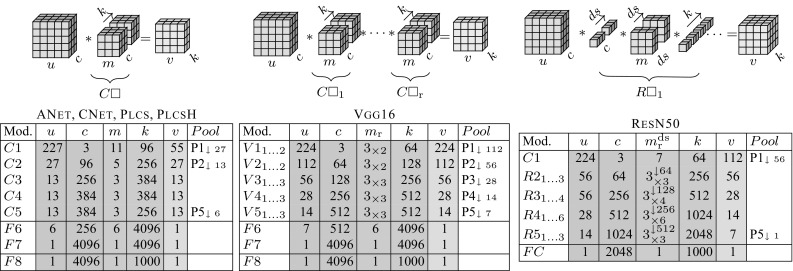
Simplified structure of the investigated convolutional neural networks. Each row of the table corresponds to a single block visualized above the table (without non-linearities, pooling and normalization layers). AlexNet (Krizhevsky et al. 2012) and its variants (Zhou et al. 2014), consist of simple convolutional layers $$C\Box $$ or fully connected layers $$F\Box $$. A block in VGG-19 network (Simonyan and Zisserman 2014) is a set of $$\mathrm {r}$$$$3\times 3$$ convolutions $$C\Box _{1\dots \mathrm {r}}$$ which operate on same spatial resolution $$u=v$$. A block of a ResN50  (He et al. 2016) networks is a set of $$\mathrm {r}$$ residual modules $$R\Box _{1\dots \mathrm {r}}$$, which consist of down-sampling, $$3\times 3$$ and up-sampling convolution (for a simplicity only a single residual module visualized). All residual modules perform on the same spatial resolution with exception of the first of a block which performs down-sampling. To keep the figure compact, we do not visualize the residual connection

Such representations are entirely handcrafted, as in the case of SIFT and HOG, or are partially learned using using proxy criteria such as *K*-means clustering, as in the case of BoVW, sparse coding, VLAD, and Fisher Vectors. In this work, HOG (Dalal and Triggs 2005) is selected as a representative of traditional image features. HOG is a variant of the SIFT descriptor which became the predominant representation in image understanding tasks before deep networks. HOG decomposes an image it into small blocks (usually of $8 \times 8$ pixels) and represents each block by a histogram of image gradient orientations. Histograms are further grouped into small partially overlapping $2 \times 2$ blocks and normalized, building invariance to illumination changes into the representation. Histograms are computed by using weighted bi-linear sampling of the image gradients, which results in approximate invariance to small image translations. Similarly, quantization of the gradient orientations and soft assignment of gradients to adjacent orientation bins gives HOG approximate invariance to small image rotations as well.

The SIFT image representation (Lowe 1999), which predates HOG, is conceptually very similar to HOG with slight differences in the normalization and gradient sampling and pooling schemes. The most significant difference is that SIFT was introduced as a descriptor of local image patches whereas HOG as a descriptor of the image as a whole, more useful for tasks such as object detection by sliding window. Due to the similarity between HOG and SIFT and due to the fact that HOG can be implemented as a small convolutional neural network (Mahendran and Vedaldi 2016), we focus on the latter in the remainder of the paper.

### Deep Learnable Image Representations

Traditional image representations have been almost entirely replaced by modern deep convolutional neural networks (CNNs). CNNs share many structural elements with representations such as HOG (which, as noted, can be implemented as as a small convolutional network); crucially, however, they are based on generic blueprints containing millions of parameters that are learned *end-to-end* to optimize the performance of the representation on a task of interest, such as image classification. As a result, these representations have dramatically superior performance than their handcrafted predecessors.

In this paper we investigate three popular families of CNNs: AlexNet-like networks (Krizhevsky et al. 2012) (ANet, CNet, Plcs  (Zhou et al. 2014)), VGG-like networks (Simonyan and Zisserman 2015) (Vgg16) and ResNet-like networks (He et al. 2016) (ResN50). Recall that a deep network is a computational chain or graph comprising operations such as linear convolution by filter banks, non-linear activation functions, pooling, and a few other simple operators. Despite differences in the local topology, AlexNet, VGG, and ResNet-like networks can generally be decomposed into a number of blocks that operate on tensors of different resolutions, with different blocks connected by down-sampling layers. This subdivision is useful to compare networks, and is summarized in Fig. 2. The performance of the selected model variants in the popular ILSVRC12 benchmark is summarized in Table 1.

**Table 1:** Performance of the selected CNN models on the ILSVRC12 dataset as implemented in Vedaldi and Lenc (2014)

	ANet (Krizhevsky et al. 2012)	CNet (Krizhevsky et al. 2012)	Vgg16 (Simonyan and Zisserman 2015)	ResN50 (He et al. 2016)
Top-5 error	19.6	19.7	9.9	7.7
Top-1 error	42.6	42.6	28.5	24.6
GFLOPs	0.727	0.724	16	4

The computational complexity is approximated in Giga-float operations per image based on Canziani et al. (2016) as measured in Albanie (2017)

In more detail, ANet is the composition of twenty functions, grouped into five convolutional layers (implementing linear filtering, max-pooling, normalization and ReLU operations) and three fully-connected layers (linear filtering and ReLU). In the paper, we analyze the output of convolution layers C1–C5, pooling layers P1, P2, P5, and of the fully connected layers F5 and F7. Features are taken immediately after the application of the linear filters (i.e. before the ReLU) and can be positive or negative, except for P1-5, which are taken *after* the non-linearity and are non-negative. We also consider the CNet variant of ANet due to its popularity in applications; it differs from ANet only slightly by placing the normalization operator before max pooling.

While ANet contains filters of various sizes, the C3–C5 layers all use $3 \times 3$ filters only. This design decision was extended in the Vgg16 model to include all convolutional layers. Vgg16 consists of 5 blocks *V*1-*V*5, each of which comprises a number of $3\times 3$ convolutional layers configured to preserve the spatial resolution of the data within a block. Max-pooling operators reduce the spatial resolution between blocks. Similarly to ANet, Vgg16 terminates in 3 fully connected layers. This network has been widely used as a plug-and-play replacement of ANet due to its simplicity and superior performance (Girshick et al. 2014a; He et al. 2014; Long et al. 2015). As with the ANet, in our experiments we consider outputs of the last convolution of the block ($V1_2 \ldots V5_3$), pooling layers P1–P5 and the fully connected layers F6 and F7.

The ResNet (He et al. 2016) architectures depart from ANet more substantially. The most obvious difference is that they contain a significantly larger number of convolutional layers. Learning such deep networks is made possible by the introduction of *residual configurations* where the input of a set of linear convolutions is added back to their outputs. ResNet also differs from ANet by the use of a single fully connected layer which performs image classification at the very end of the model; all other layers are convolutional, with the penultimate layer followed by average pooling. Conceptually, the lack of the fully connected layers is similar to the Google Inception network (Szegedy et al. 2015). This architectural difference makes ResNet slightly harder to use as a plug-in replacement for ANet in some applications (Ren et al. 2017), but the performance is generally far better than ANet and Vgg16.

We consider a single ResNet variant, ResN50. This model is organized into residual blocks, each comprising several residual units with three convolutional layers, performing dimensionality reduction, $3 \times 3$ convolution, and dimensionality expansion respectively. In our experiments, we consider outputs of six blocks, the first one *C*1 comprising a standard convolutional layer, and five residual blocks *R*2–*R*6 with a $2\times$ down-sampling during the first convolutional operation of its first (e.g. $R2_1$) with a stride-2 convolution which performs dimensionality reduction. More details about this architecture and the operations performed in each block can be found in He et al. (2016).

## Properties of Representations

So far a representation $\phi$ has been described as a function mapping an image to a vector. The design of representations is empirical, guided by intuition and validation of the performance of the representation on tasks of interest, such as image classification. Deep learning has partially automated this empirical design process by optimizing the representation parameters directly on the final task, in an end-to-end fashion.

Although the performance of representations has improved significantly as a consequence of such research efforts, we still do not understand them well from a theoretical viewpoint; this situation has in fact deteriorated with deep learning, as the complexity of deep networks, which are learned as black boxes, has made their interpretation even more challenging. In this paper we aim to shed some light on two important properties of representations: equivariance (Sect. 4.1) and equivalence (Sect. 4.2).

### Equivariance

A popular principle in the design of representations is the idea that a representation should extract from an image information which is useful for interpreting it, for example by recognizing its content, while removing the effect of *nuisance factors* such as changes in viewpoint or illumination that change the image but not its interpretation. Often, we say that a representation should be *invariant* to the nuisance factors while at the same time being *distinctive* for the information of interest (a constant function is invariant but not distinctive).

In order to illustrate this concept, consider the effect on an image $\mathbf {x}$ of certain *transformationsg* such as rotations, translations, or re-scaling. Since in almost all cases the identity of the objects in the image would not be affected by such transformations, it makes sense to seek a representation $\phi$ which is invariant to the effect of *g*, i.e. $\phi (\mathbf {x}) = \phi (g \mathbf {x})$.^2^ This notion of invariance, however, requires closure with respect to the transformation group *G* (Vedaldi and Soatto 2005): given any two transformations $g,g'\in G$, if $\phi (\mathbf {x}) = \phi (g \mathbf {x})$ and $\phi (g \mathbf {x}) = \phi (g' g\mathbf {x})$, then $\phi (\mathbf {x}) = \phi (gg'\mathbf {x})$ for the combined transformation $gg'$. Due to the finite resolution and extent of digital images, this is not realistic even for simple transformations—for example, if $\phi$ is invariant to any scaling factor $g\not = 1$, it must be invariant to any multiple $g^n$ as well, even if the scaled image $g^n\mathbf {x}$ reduces to a single pixel. Even disregarding finiteness issues, many simple transformations close onto 2D diffeomorphisms, resulting in representations that in principle should not distinguish even heavily distorted versions of the same image. In practice, therefore, invariance is often relaxed to *insensitivity to bounded transformations*: $\Vert \phi (g\mathbf {x}) -\phi (\mathbf {x})\Vert \le \epsilon \Vert g\Vert$, where $\Vert g\Vert$ is a measure of the size of the transformation.

A more fundamental problem with invariance is that the definition of a nuisance factor depends on the task at hand, whereas a representation should be useful for several tasks (otherwise there would be no difference between representations and solutions to a specific problem). For example, recognizing objects may be invariant to image translations and rotations, but localizing them clearly is *not*. Rather than removing factors of variation, therefore, often one seeks for representations that *untangle* such factors, which is sufficient to simplify the solution of specific problems while preventing others from being solved as well.

Thus, generalizing the concept of invariance, we aim at studying the equivariant properties of representations. A representation $\phi$ is *equivariant* with a transformation *g* of the input image if the transformation can be transferred to the representation output. Formally, equivariance with *g* is obtained when there exists a map $M_g : \mathbb {R}^d \rightarrow \mathbb {R}^d$ such that:

1 $$\begin{aligned} \forall \mathbf {x}\in {\mathcal {X}} : \quad \phi (g\mathbf {x}) \approx M_g \phi (\mathbf {x}). \end{aligned}$$

A *sufficient condition* for the existence of $M_g$ is that the representation $\phi$ is *invertible*, because in this case $M_g = \phi \circ g \circ \phi ^{-1}$. It is known that representations such as HOG are at least approximately invertible (Vondrick et al. 2013). Hence it is not just the existence, but also the structure of the mapping $M_g$ that is of interest. In particular, $M_g$ should be *simple*, for example a linear function. This is important because the representation is often used in simple predictors such as linear classifiers, or in the case of CNNs, is further processed by linear filters. Furthermore, by requiring the *same* mapping $M_g$ to work for *any* input image, intrinsic geometric properties of the representations are captured. Invariance is a special case of equivariance obtained when $M_g$ (or a subset of $M_g$) acts as the simplest possible transformation, i.e. the identity map.

The nature of the transformation *g* is in principle arbitrary; in practice, in this paper we will focus on geometric transformations such as affine warps and flips of the image.

As an illustrative example of equivariance, let $\phi$ denote the HOG (Dalal and Triggs 2005) feature extractor. In this case $\phi (\mathbf {x})$ can be interpreted as a $H \times W$ vector field of of *D*-dimensional feature vectors, called “cells” in the HOG terminology. If *g* denotes image flipping around the vertical axis, then $\phi (\mathbf {x})$ and $\phi (g\mathbf {x})$ are related by a well defined *permutation* of the feature components. This permutation swaps the HOG cells in the horizontal direction and, within each HOG cell, swaps the components corresponding to symmetric orientations of the gradient. Hence the mapping $M_g$ is a permutation and one has *exactly*$\phi (g\mathbf {x}) = M_g \phi (\mathbf {x})$. The same is true for horizontal flips and $180^{\circ }$ rotations, and, approximately,^3^ for $90^{\circ }$ rotations. HOG implementations (Vedaldi and Fulkerson 2010) do in fact explicitly provide such permutations.

As another remarkable example of equivariance, note that HOG, densely-computed SIFT (DSIFT), and convolutional networks are all *convolutional representations* in the sense that they are local and translation invariant operators. Barring boundary and sampling effects, convolutional representations are equivariant to translations of the input image by design, which transfer to a corresponding translation of the resulting feature field.

In all such examples, the map $M_g$ is linear. We will show empirically that this is the case for many more representations and transformations (Sect. 5).

### Covering and Equivalence

While equivariance looks at how a representation is affected by transformations of the input image, covering studies the relationship between different representations. We say that a representation $\phi$*covers* a representation $\phi '$, and we write $\phi \rightarrow \phi '$, if there exist a map $E_{\phi \rightarrow \phi '}$ such that

2 $$\begin{aligned} \forall \mathbf {x}: \quad \phi '(\mathbf {x}) \approx E_{\phi \rightarrow \phi '} \phi (\mathbf {x}). \end{aligned}$$

Covering captures the idea that $\phi$ contains at least as much information as $\phi '$. Algebraically, covering is a transitive and reflexive relation; however, it is a pre-order rather than a partial order because $\phi ' \rightarrow \phi$ and $\phi \rightarrow \phi '$ do not imply that $\phi$ and $\phi '$ are identical (i.e. the $\rightarrow$ relation is reflexive and transitive but not anti-symmetric); rather, in this case we say that they are *equivalent*, as they both carry the same information.

Note that, if $\phi$ is invertible, then $E_{\phi \rightarrow \phi '} = \phi ' \circ \phi ^{-1}$ satisfies this condition; hence, as for the mapping $M_g$ before, the interest is not just in the existence but also in the structure of the mapping $E_{\phi \rightarrow \phi '}$.

The reason why covering and equivalence are interesting properties to test for is that there exist a large variety of different image representations. In fact, each time a deep network is learned from data, the non-convex nature of the optimization results in a *different* and, as we will see, *seemingly incompatible* neural networks. However, as it may be expected, these differences are not fundamental and this can be demonstrated by the existence of simple mapping $E_{\phi \rightarrow \phi '}$ that bridge them. More interestingly, covering and equivalence can be used to assess differences in the representations computed at different depths in a neural network, as well as to compare different architectures (Sect. 6).

## Analysis of Equivariance

Given an image representation $\phi$, we study its equivariance properties (Sect. 4.1) empirically by *learning* the map $M_g$ from data. The approach, based on a structured sparse regression method (Sect. 5.1), is applied to the analysis of both traditional and deep image representations in Sects. 5.2 and 5.3, respectively. Section 5.4 shows also a practical application of these equivariant mappings to object detection using structure-output regression.

The key finding from these experiments are that:

- HOG, our representative traditional feature extractor, has a high degree of equivariance with similarity transformations (translation, rotation, flip, scale) up to limitations due to sampling artifacts.
- Deep feature extractors such as ANet, Vgg16, and ResN50 are also highly equivariant up to layers that still preserve sufficient spatial resolution, as those better represent geometry. This is also consistent with the fact that such features can be used to perform geometric-oriented tasks, such as object detection in R-CNN and related methods.
- We also show that equivariance in deep feature extractors reduces to invariance for those transformations such as left-right flipping that are present in data or in data augmentation during training. This effect is more pronounced as depth increases.
- Finally, we show that simple reconstruction metrics such as the Euclidean distance between features are not necessarily predictive of classification performance; instead, using a task-oriented regression method learns better equivariant maps in most cases.

### Methods

As our goal is to study the equivariance properties of a given image representation $\phi$, the equivariant map $M_g$ of Sect. 4.1 is not available a-priori and must be *estimated* from data, if it exists. This section discusses a number of methods to do so. First, the learning problem is discussed in general (Sect. 5.1.1) and suitable regularisers are proposed (Sect. 5.1.2). Then, efficient versions of the loss (Sect. 5.1.3) and of the map $M_g$ (Sect. 5.1.4) are given for the special case of CNN representations.

#### Learning Equivariance

Given a representation $$\phi $$ and a transformation *g*, the goal is to find a mapping $$M_g$$ satisfying (1). In the simplest case $$M_g = (A_g, \mathbf {b}_g),$$$$A_g\in \mathbb {R}^{d\times d},$$$$\mathbf {b}_g\in \mathbb {R}^d$$ is an affine transformation $$\phi (g\mathbf {x}) \approx A_g \phi (\mathbf {x}) + \mathbf {b}_g$$. This choice is not as restrictive as it may initially seem: in the examples of Sect. 4.1$$M_g$$ is a permutation, and hence can be implemented by a corresponding permutation matrix $$A_g$$.

Estimating $(A_g,\mathbf {b}_g)$ can be formulated as an empirical risk minimization problem. Given images $\mathbf {x}_1,\dots ,\mathbf {x}_n$ sampled from a set of natural images, learning amounts to optimizing the regularized reconstruction error

3 $$\begin{aligned} E(A_g,\mathbf {b}_g) = \lambda \mathcal {R}(A_g) + \frac{1}{n} \sum _{i=1}^n \ell (\phi (g\mathbf {x}_i), A_g \phi (\mathbf {x}_i) + \mathbf {b}_g), \end{aligned}$$

where $\mathcal {R}$ is a regularizer and $\ell$ a regression loss.

The choice of regularizer is particularly important as $A_g \in \mathbb {R}^{d\times d}$ has a $\varOmega (d^2)$ parameters. Since *d* can be quite large (for example, in HOG one has $d = DWH$), regularization is essential. The standard $l^2$ regularizer $\Vert A_g\Vert _F^2$ was found to be inadequate; instead, sparsity-inducting priors work much better for this problem as they encourage $A_g$ to be similar to a permutation matrix.

#### Regularizer

We consider two such sparsity-inducing regularisers. The first regularizer allows $A_g$ to contain a fixed number *k* of non-zero entries in each row:

4 $$\begin{aligned} \mathcal {R}_k(A) = {\left\{ \begin{array}{ll} +\infty , &{} \exists i: \Vert A_{i,:} \Vert _0 > k, \\ \Vert A\Vert _F^2, &{} \text {otherwise}. \end{array}\right. } \end{aligned}$$

Regularizing rows independently reflects the fact that each row is a predictor of a particular component of $\phi (g\mathbf {x})$.

**Fig. 3:**
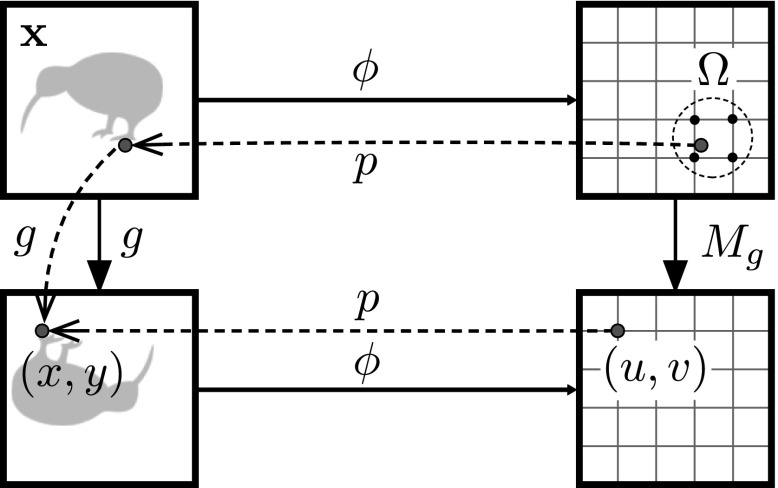
Structured sparsity. Predicting equivariant features at location (*u*, *v*) uses a corresponding small neighborhood of features $\varOmega _{g,m}(u,v)$

The second sparsity-inducing regularizer is similar, but exploits the *convolutional* structure of a representation. Convolutional features are obtained from translation invariant and local operators (non-linear filters). In this case, the representation $[\phi (\mathbf {x})]_{uvt}$ can be interpreted as a feature field or tensor with spatial indexes (*u*, *v*) and feature channel index *t*. Due to the locality of the representation, the component (*u*, *v*, *t*) of $\phi (g\mathbf {x})$ should be predictable from a corresponding neighborhood $\varOmega _{g,m}(u,v)$ of features in tensor $\phi (\mathbf {x})$ (see Fig. 3). This results in a particular sparsity structure for $A_g$ that can be imposed by the regularizer

5 $$\begin{aligned} \mathcal {R}_{g,m}(A)= {\left\{ \begin{array}{ll} +\infty , &{}\exists t, t', (u,v), (u',v') \not \in \\ &{}\varOmega _{g,m}(u,v) : A_{uvt,u'v't'}\not =0 \\ \Vert A\Vert _F^2, &{}\text {otherwise,} \\ \end{array}\right. } \end{aligned}$$

where *m* denotes the neighbor size and the indexes of *A* have been identified with triplets (*u*, *v*, *t*). The neighborhood itself is defined as the $m \times m$ input feature locations closer to the back-projection of the output feature (*u*, *v*).^4^ In practice (4) and (5) will be combined in order to limit the number of regression coefficients activated in each neighborhood.

#### Loss and Optimization

As will be shown empirically in Sect. 5.3, the choice of loss $\ell$ in Eq. (3) is important. For HOG and similar histogram-like representations, a *regression loss* such as $l^2$, Hellinger, or $\chi ^2$ distance works well. Such a loss can also be applied to convolutional architectures, although an *end-to-end task-oriented* loss can perform better. The $l^2$ loss can be easily optimized offline, for which we use a direct implementation of least squares or ridge regression, or the implementation by Sjöstrand et al. (2018) of the forward-selection algorithm. Alternatively, for CNNs the Siamese architecture approach described next works well.

**Fig. 4:**
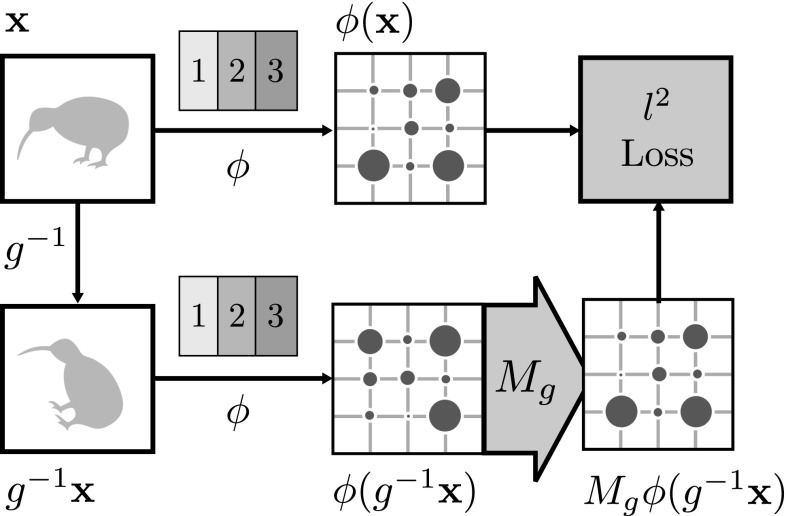
Siamese architecture for training a CNN Equivariance map. The equivariance map $M_{g^{-1}}$ aims to transform the features to minimize $l^2$ loss in the feature space. All parameters of the network are kept unchanged

**Siamese architecture for the**$l^2$**loss** For CNN representations and regression losses such as $l^2$, the transformation $M_g$ can also be learned using a Siamese architecture (Bromley et al. 1994). This is illustrated in Fig. 4: one branch of the network computes the representation of the original image $\phi (\mathbf {x})$ and the second branch computes the representation of $\psi (M_g \circ \phi (g^{-1} \mathbf {x}))$ while minimizing the $l^2$ loss between these two representations.

The Siamese approach has several advantages. First, it allows to learn $M_g$ using the same methods used to learn the CNN, usually on-line SGD optimization, which may be more memory efficient than off-line solvers. Additionally, a Siamese architecture is more flexible. For example, it is possible to apply $M_g$ after the output of a convolutional layer, but to compute the $l^2$ loss *after* the ReLU operator is applied to the output of the latter. In fact, since ReLU removes the negative components of the representation in any case, reconstructing accurately negative levels may be overkill; the Siamese configuration allows us to test this hypothesis.

**End-to-end loss** In practice, it is unclear whether a regression loss such as $l^2$ captures well the informative content of the features or whether a different metric should be used instead. In order to sidestep the issue of choosing a metric, we propose to measure the quality of feature reconstruction based on whether the features can still solve the original task.

To this end, consider a CNN $\zeta$ trained end-to-end on a categorization problem such as the ILSVRC 2012 image classification task (ILSVRC12) (Russakovsky et al. 2015). It is common (Chatfield et al. 2014; Donahue et al. 2013; Razavian et al. 2014) to consider the first several layers $\phi$ of the network $\zeta = \psi \circ \phi$ as a general-purpose feature extractor and the last layers $\psi$ as a classifier using such features. This suggests an alternative objective that preserves the quality of the features $\phi$ in the original problem:

6 $$\begin{aligned} E(A_g,\mathbf {b}_g)= & {} \lambda \mathcal {R}(A_g)\nonumber \\&+ \frac{1}{n} \sum _{i=1}^n \ell (y_i, \psi \circ (A_g,\mathbf {b}_g) \circ \phi (g^{-1} \mathbf {x}_i)). \end{aligned}$$

**Fig. 5:**
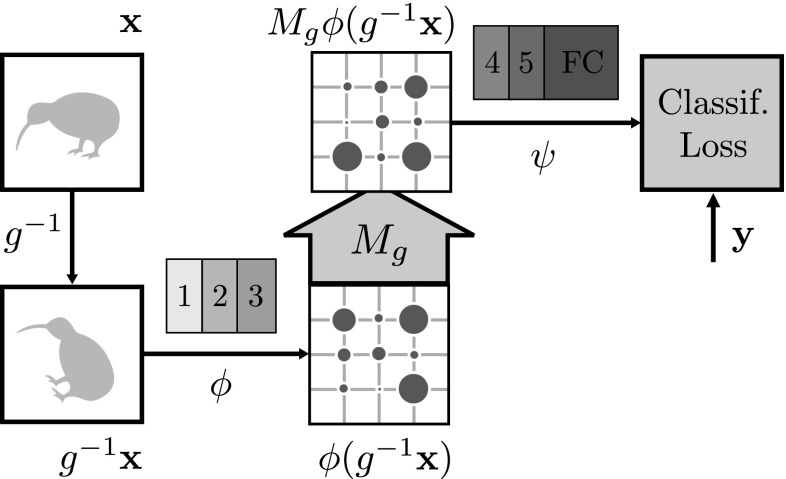
Finding equivariance map for a CNN representation using target-oriented loss. The original network (first row) is divided into the $\phi$ (feature representation) and $\psi$ (a classifier). The aim of the equivariance map $M_{g^{-1}}$ is to remove the nuisance transformation *g* in the feature space by minimizing the classification loss on the ILSVRC12 train dataset while keeping the network weights fixed

Here $y_i$ denotes the ground truth label of image $\mathbf {x}_i$ and $\ell$ is the same classification loss used to train $\zeta$. Note that in this case $(A_g,\mathbf {b}_g)$ is learned to *compensate* for the image transformation, which therefore is set to $g^{-1}$. This formulation is not restricted to CNNs, but applies to any representation $\phi$ given a target classification or regression task and a corresponding pre-trained classifier $\psi$ using it. This approach is further illustrated in Fig. 5.

**Implementation** For implementation convenience, the Siamese formulations are optimized using the same online stochastic gradient descent algorithm and weight decay used to learn the neural networks in the first place. Learning uses the MatConvNet framework (Vedaldi and Lenc 2014). The transformation layer is implemented with a layer similar to a spatial transformer (Jaderberg et al. 2015) with a fixed sampling grid. The spatial transformation and convolution with $F_g$ has little influence on the network training speed.

#### Transformation Layer

The method of Sect. 5.1 can be substantially refined for the case of CNN representations and certain classes of transformations. In fact, the structured sparsity regularizer of (5) encourages $A_g$ to match the convolutional structure of the representation. If *g* is an affine transformation more can be said: up to sampling artifacts, the equivariant transformation $M_g$ is local and translation invariant, *i.e.* convolutional. The reason is that an affine transformation *g* acts uniformly on the image domain^5^ so that the same is true for $M_g$. This has two key advantages: it dramatically reduces the number of parameters to learn and it can be implemented efficiently as an additional layer of a CNN.

Such a *transformation layer* consists of a *permutation layer*, which implements the multiplication by a permutation matrix $P_g$ moving input feature sites (*u*, *v*, *t*) to output feature sites (*g*(*u*, *v*), *t*), followed by convolution with a bank of *D* linear filters and scalar biases $(F_g, \mathbf {b}_g)$, each of dimension $m \times m \times D$. Here *m* corresponds to the size of the neighborhood $\varOmega _{g,m}(u,v)$ described in Sect. 5.1. Intuitively, the main purpose of these filters is to permute and interpolate feature channels.

Note that *g*(*u*, *v*) does not, in general, fall at integer coordinates. To address this issue, the permutation layer $P_g$ distributes *g*(*u*, *v*) to the nearest $2\times 2$ sites using bi-linear interpolation.^6^ The transformation layers allows to rewrite the learning objective as:

7 $$\begin{aligned} E(F_g,\mathbf {b}_g)= & {} \lambda \mathcal {R}(F_g) \nonumber \\&+ \frac{1}{n} \sum _{i=1}^n \ell (y_i, \psi ( F_g *( P_g \cdot \phi (g^{-1} \mathbf {x}_i) \nonumber \\&+ \mathbf {b}_g ))). \end{aligned}$$

### Results on Traditional Representations

This section applies the methods of Sect. 5.1 to learn equivariant maps for shallow representations, and HOG features in particular. The first method to be evaluated is sparse regression (Sect. 5.2.1) followed by structured sparsity (Sect. 5.2.2). A qualitative evaluation is given in Sect. 5.2.3.

**Fig. 6:**
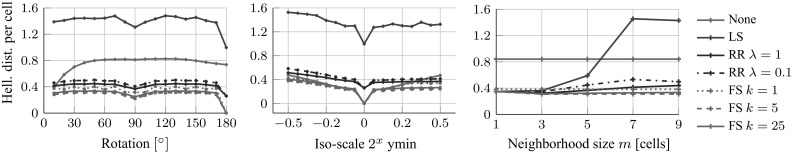
Regression methods. The figure reports the HOG feature reconstruction error (average per-cell Hellinger distance) achieved by the learned equivariant mapping $M_g$ by setting *g* to different image rotations (left) and scalings (center) for different learning strategies (see text). No other constraint is imposed on $A_g$. In the right panel the experiment is repeated for the $45^\circ$ rotation, but this time imposing structured sparsity on $A_g$ for different values of the neighborhood size *m*

#### Sparse Regression

The first experiment (Fig. 6) explores variants of the sparse regression formulation of Eq. (3). The goal is to learn a mapping $M_g=(A_g,\mathbf {b}_g)$ that predicts the effect of selected image transformations *g* on the HOG features of an image. For each transformation, the mapping $M_g$ is learned from 1000 training images by minimizing the regularized empirical risk (6). The performance is measured as the average Hellinger’s distance $\Vert \phi (g\mathbf {x})-M_g\phi (\mathbf {x})\Vert _\text {Hell.}$ on a test set of further 1000 images.^7^ Images are randomly sampled from the ILSVRC12 train and validation datasets respectively.

This experiment focuses on predicting a small array of $5\times 5$ of HOG cells, which allows to train full regression matrices even with naive baseline regression algorithms. Furthermore, the $5\times 5$ array is predicted from a larger $9 \times 9$ input array to avoid boundary issues when images are rotated or re-scaled. Both these restrictions will be relaxed later. Figure 6 compares the following methods to learn $M_g$: choosing the identity transformation $M_g=\mathbf {1}$, learning $M_g$ by optimizing the objective (3) without regularization (Least Square – LS), with the Frobenius norm regularizer for different values of $\lambda$ (Ridge Regression—RR), and with the sparsity-inducing regularizer (4) (Forward-Selection—FS, using (Sjöstrand et al. 2018)) for a different number *k* of regression coefficients per output dimension.

As can be seen in Fig. 6, LS over-fits badly, which is not surprising given that $M_g$ contains 1M parameters even for these small HOG arrays. RR performs significantly better, but it is easily outperformed by FS, confirming the very sparse nature of the solution (e.g. for $k=5$ just 0.2% of the 1M coefficients are non-zero). The best result is obtained by FS with $k=5$. As expected, the prediction error of FS is zero for a $180^{\circ }$ rotation as this transformation is exact (Sect. 5.1), but note that LS and RR fail to recover it. As one might expect, errors are smaller for transformations close to identity, although in the case of FS the error remains small throughout the range.

#### Structured Sparse Regression

**Fig. 7:**
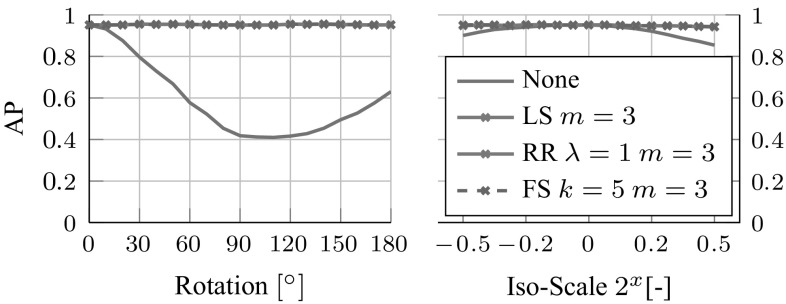
Equivariant classification using HOG features. Classification performance of a HOG-based classifier trained to discriminate dog and cat heads as the test images are gradually rotated and scaled and the effect compensated by equivariant maps learned using LS, RR, and FS

The conclusion of the previous experiments is that sparsity is essential to achieve good generalization. However, learning $M_g$ directly, e.g. by forward-selection or by $l^1$ regularization, can be quite expensive even if the solution is ultimately sparse. Next, we evaluate using the *structured sparsity* regularizer of Eq. (5), where each output feature is predicted from a pre-specified neighborhood of input features dependent on the image transformation *g*. The right plot of Fig. 6 repeats the experiment for a $45^{\circ }$ rotation, but this time limited to neighborhoods of $m \times m$ input HOG cells. To be able to span larger intervals of *m*, an array of $15 \times 15$ HOG cells is used. Since spatial sparsity is now imposed *a-priori*, LS, RR, and FS perform nearly equivalently for $m\le 3$, with the best result achieved by FS with $k=5$ and a small neighborhood of $m = 3$ cells. There is also a significant computational advantage in structured sparsity (Table 2) as it limits the effective size of the regression problems to be solved. We conclude that structured sparsity is highly preferable over generic sparsity (Fig. 7).

**Table 2:** Regression cost. Cost (in s) of learning the equivariant regressors of Fig. 7

*k*	*m*	HOG size			
		$3 \times 3$	$5 \times 5$	$7 \times 7$	$9 \times 9$
5	$\infty$	1.67	12.21	82.49	281.18
5	1	0.97	2.06	3.47	5.91
5	3	1.23	3.90	7.81	13.04
5	5	1.83	7.46	17.96	30.93

As the size of the HOG arrays becomes larger, the optimization cost increases significantly unless structured sparsity is considered by setting *m* to a small number

**Fig. 8:**
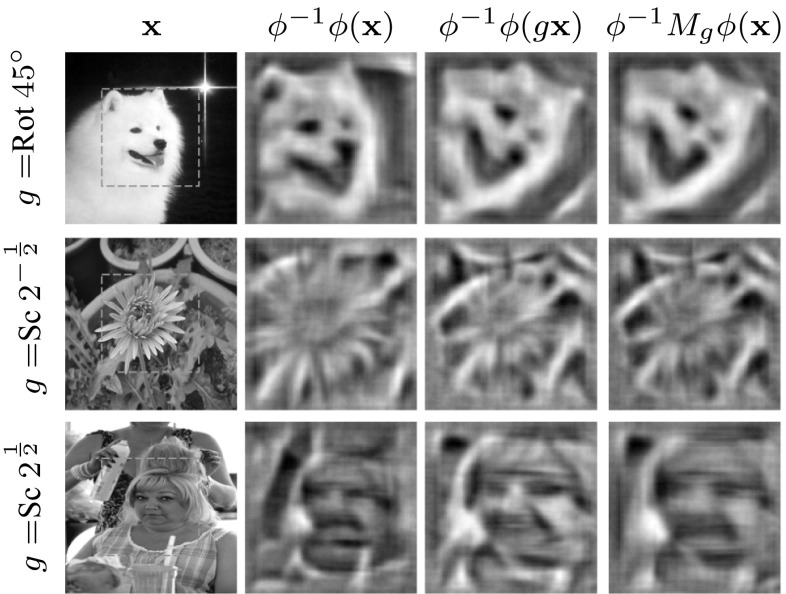
Qualitative evaluation of equivariant HOG. Visualization of the features $\phi (\mathbf {x})$, $\phi (g\mathbf {x})$ and $M_g\phi (\mathbf {x})$ using the $\phi ^{-1}$ HOGgle (Vondrick et al. 2013) HOG inverse. $M_g$ is learned using FS with $k=5$ and $m=3$ and *g* is set to a rotation by $45^{\circ }$ and up/down-scaling by $\sqrt{2}$ respectively. The dashed boxes show the support of the reconstructed features

**Fig. 9:**
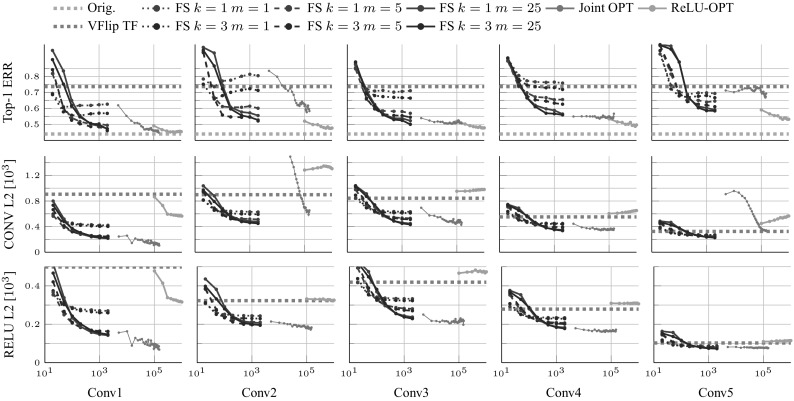
Comparison of regression methods for a CNN. Regression error of an equivariant map $M_g$ learned for vertical image flips for different layers of a CNN. FS (gray and brown lines) and the task-oriented objective (purple) are evaluated against the number of training samples. Both the task loss (top) and the feature reconstruction error (bottom) are reported. In the task loss, the green dashed line is the performance of the original classifier on the original images (best possible performance) and the red dashed line the performance of this classifier on the transformed images (worst case). In the second row, the $l^2$ reconstruction error per cell is visualized together with the baseline—average $l^2$ distance of the representation to zero vector (Color figure online)

#### Regression Quality

So far results have been given in term of the reconstruction error of the features; this paragraph relates this measure to the practical performance of the learned mappings. The first experiment is qualitative and uses the HOGgle technique (Vondrick et al. 2013) to visualize the transformed features. As shown in Fig. 8, the visualizations of $\phi (g\mathbf {x})$ and $M_g \phi (\mathbf {x})$ are indeed nearly identical, validating the mapping $M_g$. The second experiment (Fig. 7) evaluates instead the performance of transformed HOG features quantitatively, in a classification problem. To this end, an SVM classifier $\langle \mathbf {w}, \phi (\mathbf {x}) \rangle$ is trained to discriminate between dog and cat faces using the data of Parkhi et al. (2011) (using $15\times 15$ HOG templates, 400 training and 1000 testing images evenly split among cats and dogs). Then a progressively larger rotation or scaling $g^{-1}$ is applied to the input image and the effect compensated by $M_g$, computing the SVM score as $\langle \mathbf {w}, M_g \phi (g^{-1}\mathbf {x}) \rangle$ (equivalently the model is transformed by $M_g^\top$). The performance of the compensated classifier is nearly identical to the original classifier for all angles and scales, whereas the uncompensated classifier $\langle \mathbf {w}, \phi (g^{-1}\mathbf {x})\rangle$ rapidly fails, particularly for rotation. We conclude that equivariant transformations encode visual information effectively.

### Results on Deep Representations

This section extends the experiments of the previous section on deep representations, including investigations with task-oriented losses.

#### Regression Methods

In this section we validate the parameters of various regression methods and show that the task-oriented loss results in better equivariant maps.

The first experiment (Fig. 9) compares different methods to learn equivariant mappings $M_g$ in a CNN. The first method (gray and brown lines) is FS, computed for different neighborhood sizes *k* (line color) and sparsity *m* (line pattern). The next method (blue line) is the $l^2$ loss training after the ReLU layer, as specified in Sect. 5.1.3. The last method (orange line) is the task oriented formulation of Sect. 5.1 using a transformation layer.

The classification error (task-oriented loss, first row), $l^2$ reconstruction error (second row) and $l^2$ reconstruction error after the ReLU operation (third row) are reported against the number of training samples seen. As in Sect. 5.1.4, the latter is the classification error of the compensated network $\psi \circ M_g \circ \phi (g^{-1}\mathbf {x})$ on ImageNet ILSVCR12 data (the reported error is measured on the validation data, but optimized on the training data). The figure reports the evolution of the loss as more training samples are used. For the purpose of this experiment, *g* is set to be vertical image flipping. Figure 11 repeats the experiments for the task-oriented objective and rotations *g* from 0 to 90 degrees (the fact that intermediate rotations are slightly harder to reconstruct suggests that a better $M_g$ could be learned by addressing more carefully interpolation and boundary effects).

Several observations can be made. First, all methods perform substantially better than doing nothing (which has $75\%$ top-1 error, red dashed line), recovering most if not all the performance of the original classifier ($43\%$, green dashed line). This demonstrates that linear equivariant mappings $M_g$ can be learned successfully for CNNs too. Second, for the shallower features up to C2, FS is better: it requires less training samples (as it uses an offline optimizer) and it has a smaller reconstruction error and comparable classification error than the task-oriented loss. Compared to Sect. 5.2, however, the best setting $m=3$, $k=25$ is substantially less sparse. From C3 onward, the task-oriented loss is better, converging to a much lower classification error than FS. FS still achieves a significantly smaller reconstruction error, showing that feature reconstruction is not always predictive of classification performance. Third, the classification error increases somewhat with depth, matching the intuition that deeper layers contain more specialized information: as such, perfectly transforming these layers for transformations which were not experienced during training (e.g. vertical flips) may not be possible.

Because the CNN uses a ReLU non-linearity, one can ask whether optimizing the $l^2$ loss before the non-linearity is apt for this task. To shed light on this question, we train $M_g$ using a $l^2$ loss after the non-linearity (ReLU-OPT). One can see that this still performs slightly worse than the task-specific loss, even though it performs slightly better than the FS (which may be due to more training data). However it is interesting to observe that neither the $l^2$ loss before or after the non-linearity is strongly predictive of the target performance. Thus we conclude that the $l^2$ metric should only be used as a proxy metric in the hidden representation of the CNNs (with respect to the target task).

#### Comparing Transformation Types

**Fig. 10:**
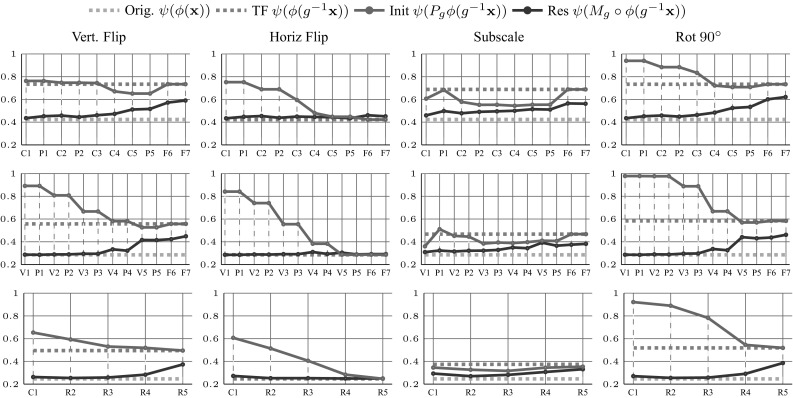
Equivariance of various networks to selected transformations. Equivariance of selected network feature representations (rows) under selected transformations (columns). The green dashed line is the initial error rate of the selected network on the ILSVRC12 validation dataset. The red dashed line represents error rate for transformed images. The gray solid line visualizes the initial performance by only spatially rearranging the features and the orange solid line shows the performance of the learn equivariant map $M_g$. For all networks, the representation used is the last block of the specified module

Next we investigate which geometric transformations can be represented by different layers of various CNNs (Fig. 10), considering in particular horizontal and vertical flips, re-scaling by half, and rotation of $90^{\circ }$. We perform this experiment for three CNN models. For ANet and Vgg16 the experiment is additionally performed on two of its fully connected layer representations. This is not applicable for the ResN50 which has only the final classifier as a fully connected layer. In all experiments, the training is done for five epochs of $2 \cdot 10^5$ training samples, using a constant learning rate of $10^{-2}$.

For transformations such as horizontal flips and scaling, learning equivariant mappings is not better than leaving the features unchanged: this is due to the fact that the CNN implicitly learns to be invariant to such factors. For vertical flips and rotations, however, the learned equivariant mapping substantially reduce the error. In particular, the first few layers for all three investigated networks are easily transformable, confirming their generic nature.

The results also show that finding an equivariant transformation for fully connected layers (or layers with lower spatial resolution in general) is more difficult than for convolutional layers. This is consistent with the fact that the deepest layers of networks contain less spatial information and hence expressing geometric transformations on top of them becomes harder. This is also consistent with the fact that ResN50 shows better equivariance properties for deeper layers compared to Vgg16 and ANet: the reason is that ResN50 preserves spatial information deeper in the architecture.

#### Qualitative Evaluation

Similarly to the visualization we obtained for the HOG features, we can use the pre-image method of Mahendran and Vedaldi (2016) to invert each deep representation and assess the learned mappings visually. Figure 10 shows the inverse of the maps $\phi (g\mathbf {x})$ and $M_g \phi (x)$ for different representations corresponding to different layers of ANet. It also shows the results obtained by inverting with $P_g \phi (\mathbf {x})$, considering only a permutation matrix $P_g$ instead of using the fully-fledged map $M_g$. In this experiment, $M_g$ is obtained using the task-oriented optimization.

We can see that in all cases the pre-images $M_g \phi (\mathbf {x})]^{-1}$ are nearly always better than the pre-images $[\phi (g\mathbf {x})]^{-1}$, which validates the equivariant map $M_g$. Furthermore, in all cases the pre-image obtained using $M_g$ is better than the one obtained using the simple permutation $P_g$, which confirms that both permutation and feature channel transformation are needed to achieve equivariance.

#### Geometric Invariances

This section explores the geometric invariance properties of different neural network architectures. This is done by measuring the performance of the hybrid network $\psi (P_g \phi (g^{-1} \mathbf {x}))$, where the spatial permutation matrix $P_g$ is used to undo the effect of the geometric transformation in feature space as was done with the task-oriented objective (7). We compare this result to the one obtained previously where $P_g$ was generalized to the learned equivariant map $M_g$: the idea is that if the spatial permutation $P_g$ is sufficient to achieve the same performance as $M_g$ then the feature channels are already invariant to the nuisance transformation.

**Fig. 11:**
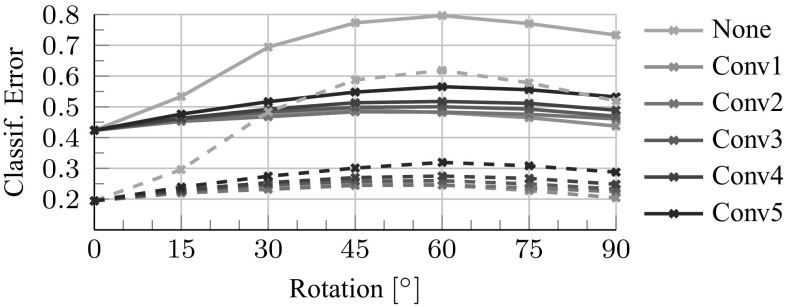
Learning equivariant CNN mappings for image rotations. The setting is similar to Fig. 9, extended to several rotations *g* but limited to the task-oriented regression method for the ANet. The solid and dashed lines report the top1 and top5 errors on the ILSVRC12 validation set respectively

**Fig. 12:**
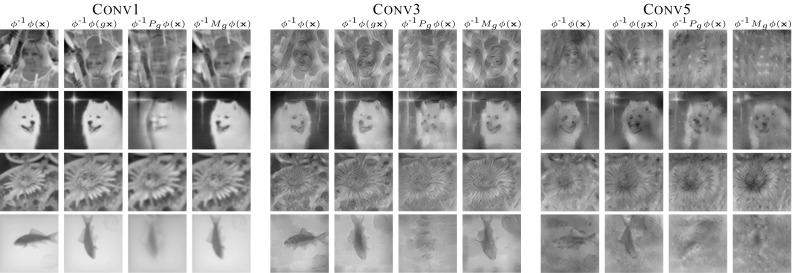
Qualitative evaluation of $M_g$. Visualization of the features $\phi (\mathbf {x})$, $\phi (g\mathbf {x})$ and $M_g\phi (\mathbf {x})$ of C1, C2 and C3 representations of ANet using the $\phi ^{-1}$ Deep Goggle (Mahendran and Vedaldi 2016) for feature inverse. Inverse of the input image and of transformed image are in the first two columns. Third column is inverse of the features with only spatially re-arranged representation with a permutation matrix $P_g$. $M_g$ is learned using the joint optimization (see quantitative results in Fig. 10) and should be ideally equal to the second column

The performance of $P_g$ against $M_g$ is visualized in Fig. 10 (gray vs orange lines) for the different layers of ANet, Vgg16, and ResN50. We note that the invariance to horizontal flips is obtained progressively with depth. Consequently,m the fully convolutional layers have access to a representation which is already invariant to this geometric transformation, which significantly simplifies the image classification task.

We also observe that there is a certain degree of scale invariance in the C5 representation of ANet and Vgg16 networks. This may help to explain why R-CNN object detectors such as (Girshick 2015; He et al. 2014; Ren et al. 2015) work well. Recall that thee methods use a simple spatial resampler such as Spatial Pyramid Pooling to extract features in correspondence of objects of different sizes and locations in the image. Resampling spatial coordinates is in principle insufficient to make the extracted region representation invariant to scale changes, unless, as it appears to be the case, the feature channel values are also insensitive to scale.

**Table 3:** CNN invariance

Layer	Horiz. Flip		Vert. Flip		Sc. $2^{-\frac{1}{2}}$		Rot. $90^{\circ }$	
	Num	%	Num	%	Num	%	Num	%
**C1**	52	54.17	53	55.21	95	98.96	42	43.75
**C2**	131	51.17	45	17.58	69	26.95	27	10.55
**C3**	238	61.98	132	34.38	295	76.82	120	31.25
**C4**	343	89.32	124	32.29	378	98.44	101	26.30
**C5**	255	99.61	47	18.36	252	98.44	56	21.88

Number and percentage of invariant feature channels in the Alexn network, identified by analyzing corresponding equivariant transformations

Additionally, it can be seen in Fig. 10, that applying only the permutation $P_g$ on the lower layers significantly reduces the performance of the network. We can observe that earlier representations are “*anti*-invariant” since the rest of the network is more sensitive to this nuisance transformation when this is applied in feature space (Figs. 11, 12).

Next, we study the map $F_g$ to identify which feature channels are invariant: these are the ones that are best predicted by themselves after a transformation. However, invariance is almost never achieved exactly; instead, the degree of invariance of a feature channel is scored as the ratio of the Euclidean norm of the corresponding row of $F_g$ with the same row after suppressing the “diagonal” component of that row. The *p* rows of $F_g$ with the highest invariance score are then replaced by (scaled) rows of the identity matrix. Finally, the performance of the modified transformation $\bar{F}_g$ is evaluated and accepted if the classification performance does not deteriorate by more than $5\%$ relative to $F_g$. The corresponding feature channels for the largest possible *p* are then considered approximately invariant.

Table 3 reports the result of this analysis for horizontal and vertical flips, re-scaling, and $90^{\circ }$ rotation in the ANet CNN. There are several notable observations. First, for transformations in which the network has achieved invariance such as horizontal flips and re-scaling. This invariance is obtained largely in C3 or C4. Second, invariance does not always increase with depth (for example C1 tends to be more invariant than C2). This is possible because, even if the feature channels within a layer are invariant, the spatial pooling in the subsequent layer may not be. Third, the number of invariant features is significantly smaller for unexpected transformations such as vertical flips and $90^{\circ }$ rotations, further validating the approach. These results corroborate the finding reported in Fig. 10, first row.

### Application to Structured-Output Regression

To complement the theoretical investigation thus far, this section shows a direct practical application of the learned equivariant mappings of Sect. 5 to the task of structured-output regression (Taskar et al. 2003). In structured regression an input image $\mathbf {x}$ is mapped to a label $\mathbf {y}$ by the function $\hat{\mathbf {y}}(\mathbf {x}) = {\text {argmax}}_{\mathbf {y},\mathbf {z}} \langle \phi (\mathbf {x},\mathbf {y},\mathbf {z}), \mathbf {w}\rangle$ (direct regression) where $\mathbf {z}$ is an optional latent variable and $\phi$ is a joint feature map. If either $\mathbf {y}$ or $\mathbf {z}$ include geometric parameters, the joint features can be partially or fully rewritten as $\phi (\mathbf {x},\mathbf {y},\mathbf {z})= M_{\mathbf {y},\mathbf {z}} \phi (\mathbf {x})$, reducing inference to the maximization of $\langle M_{\mathbf {y},\mathbf {z}}^\top \mathbf {w}, \phi (\mathbf {x})\rangle$ (equivariant regression). There are two computational advantages to this approach: (i) the representation $\phi (\mathbf {x})$ needs only to be computed once and (ii) the vectors $M_{\mathbf {y},\mathbf {z}}^\top \mathbf {w}$ can be pre-computed offline.

**Table 4:** Equivariant regression. The table reports the prediction errors for the cat head rotation/affine pose with direct/equivariant structured SVM regressors

$\phi (x)$	Bsln	HOG		C3		C4		C5	
		*g*	$M_{g}$	*g*	$M_{g}$	*g*	$M_{g}$	*g*	$M_{g}$
Rot ($^{\circ }$)	23.8	14.9	17.0	13.3	11.6	10.5	11.1	10.1	13.4
Rot $\circlearrowleft$ ($^{\circ }$)	86.9	18.9	19.1	13.2	15.0	12.8	15.3	12.9	17.4
Aff (–)	0.35	0.25	0.25	0.25	0.28	0.24	0.26	0.24	0.26
Time/TF (ms)	–	18.2	0.8	59.4	6.9	65.0	7.0	70.1	5.7
Speedup (–)	–	1	21.9	1	8.6	1	9.3	1	12.3

The error is measured in expected degrees of residual rotation or as the average keypoint distance in the normalized face frame, respectively. The baseline (denoted as Bsln) method predicts a constant transformation

This idea is demonstrated on the task of pose estimation, where $\mathbf {y}= g$ is a geometric transformation in a class $g^{-1}\in G$ of possible poses of an object. As an example, consider estimating the pose of cat faces in the PASCAL VOC 2007 (VOC07) (Everingham et al. 2007) data taking *G* either to be (i) rotations or (ii) affine transformations (Fig. 14). The rotations in *G* are sampled uniformly every 10 degrees and the ground-truth rotation of a face is defined by the line connecting the nose to the midpoints between the eyes. These keypoints are obtained as the center of gravity of the corresponding regions in the VOC07 part annotations (Chen et al. 2014). The affine transformations in *G* are obtained by clustering the vectors $[\mathbf {c}_l^\top , \mathbf {c}_r^\top ,\mathbf {c}_n^\top ]^\top$ containing the location of eyes and nose of 300 example faces in the VOC07 data.

The clusters are obtained using GMM-EM on the training data and used to map the test data to the same pose classes for evaluation. *G* then contains the set of affine transformations mapping the keypoints $[\bar{\mathbf {c}}_l^\top , \bar{\mathbf {c}}_r^\top , \bar{\mathbf {c}}_n^\top ]^\top$ in a canonical frame to each cluster center.

The matrices $M_g$ are pre-learned (from generic images not containing cats) using FS with $k=5$ and $m=3$ as in Sect. 5.1. Since cat faces in VOC07 data are usually upright, a second more challenging version of the data (denoted by the symbol $\circlearrowleft$) augmented with random image rotations is considered as well. The direct $\langle \mathbf {w}, \phi (g\mathbf {x})\rangle$ and equivariant $\langle \mathbf {w}, M_{g}\phi (\mathbf {x})\rangle$ scoring functions are learned using 300 training samples and evaluated on 300 test ones.

**Fig. 13:**
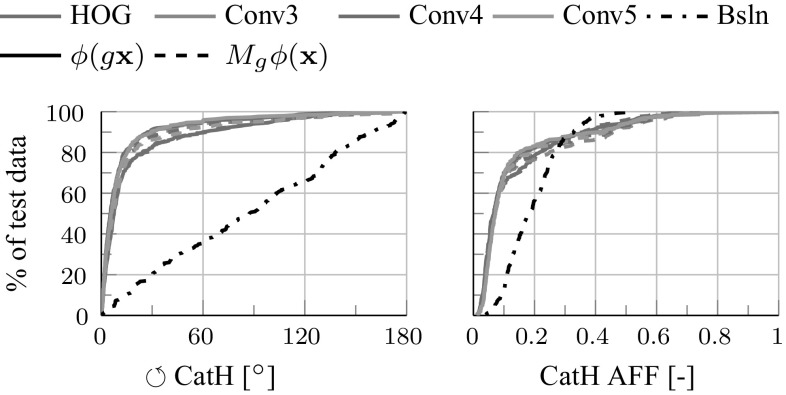
Equivariant regression errors. Cumulative error curves for the rotation and affine pose regressors of Table 4

Table 4 reports the accuracy and speed obtained for HOG and ANet CNN C3, C4, and C5 features for direct and equivariant regression. The latter is generally as good or nearly as good as direct regression, but up to 22 times faster further validating the mappings $M_g$. Figure 13 shows the cumulative error curves for the different regressors.

## Analysis of Coverage and Equivalence

We now move our attention from equivariance to coverage and equivalence of CNN representations by first adapting the methods developed in the previous section to this analysis (Sect. 6.1) and then using them to studying numerous cases of interest (Sect. 6.2).

The key finding from these experiments are that:

- Different networks trained to perform the same task tend to learn representations that are approximately equivalent.
- Deeper and larger representations tend to cover well for shallower and smaller ones, but the converse is not always true. For example, the deeper layers of ANet cover for the shallower layers of the same network, Vgg16 layers cover well for ANet layers, and ResN50 layers cover well for Vgg16 layers. However, Vgg16 layers cannot cover for ResN50 layers.
- Coverage and equivalence tend to be better for layers whose output spatial resolution matches. In fact, a layer’s resolution is a better indicator of compatibility than its depth.
- When the same network is trained on two different tasks, shallower layers tend to be equivalent, whereas deeper ones tend to be less so, as they become more task-specific.

### Methods

**Fig. 14:**
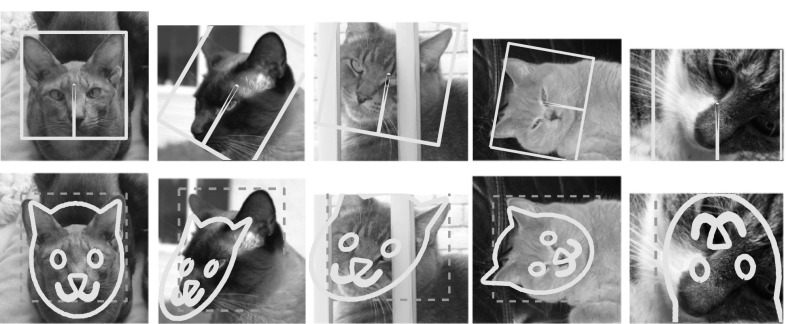
Equivariant regression examples. Rotation (top) and affine pose (bottom) prediction for cat faces in the VOC07 parts data. The estimated affine pose is represented by eyes and nose location. The first four columns contain examples of successful regressions and the last column shows a failure case. Regression uses CNN C5 features computed within the green dashed box region

As for the map $M_g$ in the case of equivariance, the covering map $E_{\phi \rightarrow \phi '}$ of  Eq. (2) must be estimated from data. Fortunately, a number of the algorithms used for estimating $M_g$ are equally applicable to $E_{\phi \rightarrow \phi '}$. In particular, the objective (3) can be adapted to the covering problem by replacing $\phi (g\mathbf {x})$ by $\phi '(\mathbf {x})$. Following the task-oriented loss formulation of Sect. 5.1, consider two representations $\phi$ and $\phi '$ and a predictor $\psi '$ learned to solve a reference task using the representation $\phi '$. For example, these could be obtained by decomposing two CNNs $\zeta = \psi \circ \phi$ and $\zeta ' = \psi ' \circ \phi '$ trained on the ImageNet ILSVRC12 data (but $\phi$ could also be learned on a different dataset, with a different network architecture or could be an handcrafted feature representation) (Fig. 14).

**Fig. 15:**
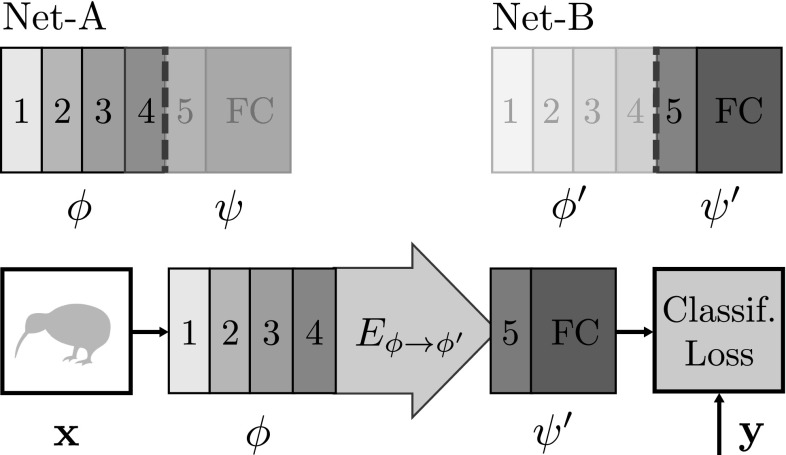
CNN architecture for learning a covering map. In order to learn the covering map $E_{\phi \rightarrow \phi '}$ we stitch the shallower layers of network Net-A (forming representation $\phi$) to the deeper layers of network Net-B (forming an image classifier $\psi '$). The stitching map $E_{\phi \rightarrow \phi '}$ is to optimized to minimize the classification loss on the training set

The goal is to find a mapping $E_{\phi \rightarrow \phi '}$ such that $\phi ' \approx E_{\phi \rightarrow \phi '} \phi$. This map can be seen as a “stitching transformation” allowing $\psi ' \circ E_{\phi \rightarrow \phi '} \circ \phi$ to perform as well as $\psi ' \circ \phi '$ on the original classification task. Hence this transformation can be learned by minimizing the loss $\ell (y_i, \psi ' \circ E_{\phi \rightarrow \phi '} \circ \phi (\mathbf {x}_i))$ with an objective similar to (6), resulting in the architecture of Fig. 15.

In a CNN, the stitching transformation $E_{\phi \rightarrow \phi '}$ can be implemented as a *stitching layer*. Given the convolutional structure of the representation, this layer can be implemented as a bank of linear filters. No permutation layer is needed in this case, but it may be necessary to down/up-sample the features if the spatial dimensions of $\phi$ and $\phi '$ do not match. This is done by using nearest neighbor interpolation for down-sampling and bilinear interpolation for up-sampling, resulting in a definition similar to (7), where $P_g$ is defined as up-scaling or down-scaling based on the spatial resolution of $\phi$ and $\phi '$.

In all experiments, training is done for seven epochs with $2\cdot 10^5$ training samples, using a constant learning rate of $10^{-2}$. The *E* map is initialized randomly with the Xavier method (Glorot and Bengio 2010), although we have observed that results are not sensitive to the form of initialization (random matrix, random permutation and identity matrix) or level of weight decay.

### Results

The goal of this experimental section is to asses whether different image representations carry similar information. We perform three different investigations: covering of representations produced by different layers of the same network (Sect. 6.2.1), covering of representations obtained by training the same CNN architecture on different tasks (Sect. 6.2.2), and covering of representations obtained from different CNN architectures (Sect. 6.2.3).

**Table 5:** Stitching different variants of the ANet architecture—mean and a standard deviation of the top1 error over 3 training runs with different random seed

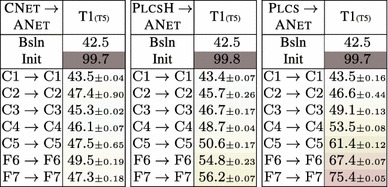

**Table 6:** Stitching different layers of the CNet network. The top1 error of the original CNet network is $42.5\%$

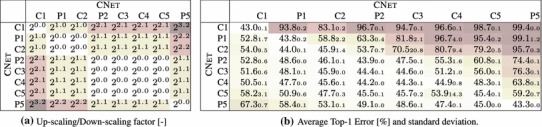	

The trained network on row *m* and column *n* is $\phi ^{Row}_1 \dots \phi ^{Row}_m \circ E \circ \phi ^{Col}_n \dots \phi ^{Col}_{\text {softmax}}$. The top-1 error is shown as a mean of 3 experiments with the standard deviation as the subscript value

#### Same Architecture, Different Layers

In the first experiment we “stitch” different layers of the same neural network architecture. This is done to assess the degree of change between different layers and to provide a baseline level of performance for subsequent experiments. Note that,x when a layer is stitched to itself, the ideal stitching transformation *E* is the identity; nevertheless, we still initialize the map *E* with a random noise and learn it from data. Due to the non-convex nature of the optimization, this will not in general recover the identity transformation perfectly, and can be used to assess the performance loss due to the limitations of the optimization procedure ((Yosinski et al. 2014) refer to this issue as “fragile co-adaptation”) (Table 5).

Table 6b shows the results of this experiment on the CNet network. We test the stitching of any pair of layers in the architecture, to construct a matrix of results. Each entry in the matrix reports the accuracy of the stitched network on the ILSVRC12 data after learning the map $E_{\phi \rightarrow \phi '}$ initialized from random noise (without learning, the error rate is 100% in all cases). There are three cases of interest: the diagonal (stitching a layer to itself), the upper diagonal (which amounts to skipping some of the layers) and the lower diagonal (which amounts to recomputing some of the layers twice).

Along the diagonal, there is a modest performance drop as a result of the fragile co-adaptation effect.

For the upper diagonal, skipping layers may reduce the network performance substantially. This is particularly true if one skips C2, but less so when skipping one or more of C3–C5. We note that C3–C5 operate on the same resolution, different to that of C2, so a portion of the drop can be explained by effects of aliasing in down-sampling the feature maps in the stitching layer.

For the lower diagonal, rerouting the information through part of the network twice tends to preserve the baseline performance. This suggests that the stitching map *E* can learn to “undo” the effect of several network layers despite being a simple linear projection. One possible interpretation is that, while layers perform complex operations such as removing the effect of nuisance factors and building invariance, it is easy to reconstruct an equivalent version of the input given the result of such operations. Note that, since deeper layers contain many more feature channels than earlier ones, the map *E* performs dimensionality reduction. Still, there are limitations: we also evaluated reconstruction of the input image pixels, but in this case the error rate of the stitched network remained $>\,94\%$.

The asymmetry of the results show the importance of distinguishing the concepts of coverage (asymmetric) and equivalence (symmetric). Our results can be summarized as follows *“the deep layers of a neural network cover the earlier layer, but not vice-versa”*.

Table 6b also reports the standard deviation of the results obtained by randomly re-initializing *E* and re-learning it several times. The stability of the results is proportional to their quality, suggesting that learning *E* is stable when stitching compatible representations and less stable otherwise.

Finally, we note that there is a correlation between the layers’ resolution and their compatibility. This can be observed in the similarity of Table 6a, reporting the resolution change, and Table 6b, reporting the performance of the stitched model. We see that there are subtle differences—e.g. for the block of P2–C5, where no sampling is performed, C5 is clearly more compatible with C4 than with P2. Similarly, down-sampling by a factor of $2^{1.1}$, can lead to a top-1 error from $59.2\%$ up to $95.4\%$. We conclude that down-sampling/up-sampling may lead to an offset in the results score, however there are still clear differences between the results obtained for the same constant factor. Thus we can use these results for drawing observations about the representation compatibility.

#### Same Architecture, Different Tasks

Next, we investigate the compatibility of nearly identical architectures trained on the same data twice, or on different data. In more detail, the first several layers $\phi '$ of the ANet CNN $\zeta '=\psi '\circ \phi '$ are swapped with layers $\phi$ from CNet, also trained on the ILSVRC12 data, Plcs  (Zhou et al. 2014), trained on the MIT Places data, and PlcsH, trained on a mixture of MIT Places and ILSVRC12 images. These representations have a similar, but not identical, structure and different parameterizations as they are trained independently.

Table 5 reports the top-1 error on ILSVRC12 of the hybrid models $\psi ' \circ E_{\phi \rightarrow \phi '} \circ \phi$ where the covering map $E_{\phi \rightarrow \phi '}$ is learned as usual. There are a number of notable facts. First, setting $E_{\phi \rightarrow \phi '}=\mathbf {1}$ to the identity map has a top-1 error $>99\%$ (not shown in the table), confirming that different representations are not directly compatible. Second, a strong level of equivalence can be established up to C4 between ANet and CNet, slightly weaker level can be established between ANet and PlcsH, and only a poor level of equivalence is observed for the deepest layers of Plcs. Specifically, the C1-2 layers of all networks are almost always interchangeable, whereas C5 is not as interchangeable, particularly for Plcs. This corroborates the intuition that C1-2 are generic image codes, whereas C5 is more task-specific. Still, even in the worst case, performance is dramatically better than chance, demonstrating that all such features are compatible to an extent. Results are also stable over repeated learning of the map *E*.

#### Different Architectures, Same Task

The final experiment assesses the equivalence between layers of different neural network architectures trained on the same data. In this case, we stitch the output of the linear convolution layers as well as the output of the pooling layers, after ReLUs. Note that, since the two architecture differ, there is no “obvious” stitching point, so each possibility is evaluated.

**Table 7:** Equivalence of ANet features with Vgg16 classifiers, top-1 error

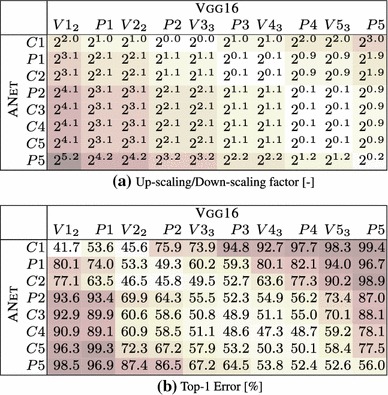	

The initial performance of ANet is $42.5\%$, which provides the theoretical upper bound of achievable performance

**ANet**$\rightarrow$**Vgg16** Table 7 shows the effect of replacing a subset of the Vgg16 layers with layers from the ANet network. Generally, the ANet can partially cover the Vgg16 layers, but there is almost always a non-negligible performance drop compared to the more powerful Vgg16 configuration. The presence of the ReLU activation functions has little to no influence on coverage.

Contrary to the previous experiment, deeper ANet features fail to cover for earlier Vgg16 features (whereas deeper ANet features can generally cover well for early ANet features). It is possible that the constrained structure of the map $E_{\phi \rightarrow \phi '}$ fails to capture the required transformation.

**Table 8:** Equivalence of Vgg16 features with ANet classifiers, top-1 error

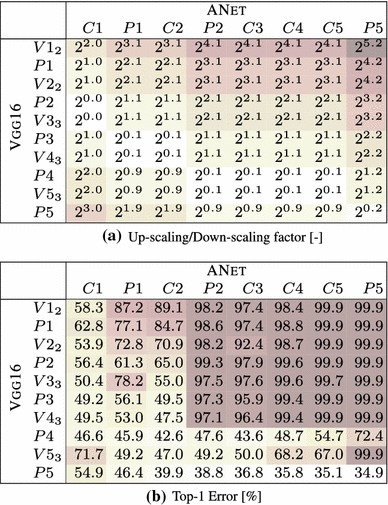	

The initial performance of Vgg16 is $28.5\%$, which is the theoretical lower bound of the achievable performance

**Vgg16**$\rightarrow$**ANet** Next, Table 8 tests the reverse direction: whether Vgg16 can cover ANet features. The answer is mixed. The output of the Vgg16*P*5 layer can cover well for ANet*C*2 to *P*5, even though there is a significant resolution change. In fact, the performance is significantly better than ANet alone, (reducing the 42.5 top-1 error of ANet to 34.9), which suggests the degree to which the representational power of Vgg16 is contained in the convolutional layers. The ability of Vgg16-P5 to cover for ANet-*C*2–*P*5 may also be explained by the fact that the last three layers of ANet have a similar structure as the *V*4 block of Vgg16, as they all use $3\times 3$ filters.

On the other hand, the earlier layers of Vgg16 cover significantly less well for ANet features than Vgg16-*P*5.

**Table 9:** Stitching ResN50 feature representations to Vgg16 classifiers

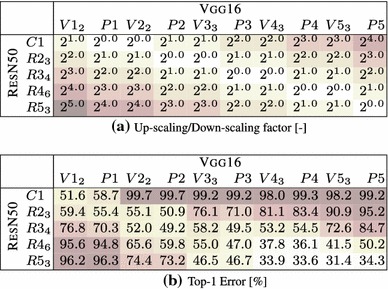	

The top-1 error of the Vgg16 network is $28.5\%$

**ResN50**$\rightarrow$**Vgg16** Next, in Table 9 we asses whether ResN50 features can cover Vgg16 features. As seen in Fig. 2, these two architectures differ significantly in their structure; consequently, ResN50 fails to cover well for Vgg16 in most cases. Good performance is however obtained by stitching the top layers; for example, ResN50-$R5_3$ covers well Vgg16-*P*5. This suggests that the final layers of ResN50 are more similar to the top convolutional layers of Vgg16 than to its fully connected layers. This indicates that the main driving factor establishing the kind of information captured at different depths is predominantly controlled by the spatial resolution of the features rather than by the depth or complexity of the representation.

Vgg16$\rightarrow$ResN50 It was not possible to use Vgg16 features to cover for ResN50 with our method at all. In all cases, the error remained $>90\%$. We hypothesize that the lack of the residual connections in the Vgg16 network makes the features incompatible with the ResN50 ones.

## Summary

This paper introduced the idea of studying representations by learning their equivariant and coverage/equivalence properties empirically. It was shown that shallow representations and the first several layers of deep state-of-the-art CNNs transform in an easily predictable manner with image warps. It was also shown that many representations tend to be interchangeable, and hence equivalent, despite differences, even substantial ones, in the architectures. Deeper layers share some of these properties but to a lesser degree, being more task-specific.

A similarity of spatial resolution is a key predictor of representations compatibility; having a sufficiently-large spatial resolution is also predictive of the equivariance properties to geometric warps. Furthermore, deeper and larger representations tend to cover well for shallower and smaller ones.

In addition the usage as analytical tools, these methods have practical applications such as accelerating structured-output regressors classifier in a simple and elegant manner.

## Footnotes

1 A common next step for these algorithms is to reduce the various hypothesis with e.g. RANSAC (Fischler and Bolles 1981) in local feature pipelines or non-maxima-suppression for object detection tasks (Dalal and Triggs 2005; Girshick et al. 2014b).

2 Here, $g:\mathbb {R}^2\rightarrow \mathbb {R}^2$ is a transformation of the plane. An image $\mathbf {x}$ is, up to discretization, a function $\mathbb {R}^2\rightarrow \mathbb {R}^3$. The action $g\mathbf {x}$ of the transformation on the image results in a new image $[g\mathbf {x}](u)=\mathbf {x}(g^{-1}(u))$.

3 Most HOG implementations use 9 orientation bins, breaking rotational symmetry.

4 Formally, denote by (*x*, *y*) the coordinates of a pixel in the input image $\mathbf {x}$ and by $p:(u,v)\mapsto (x,y)$ the affine function mapping the feature index (*u*, *v*) to the center (*x*, *y*) of the corresponding *receptive field* (measurement region) in the input image. Denote by $\mathcal {N}_k(u,v)$ the *k* feature locations $(u',v')$ that are closer to (*u*, *v*) (the latter can have fractional coordinates) and use this to define the neighborhood of the back-transformed location (*u*, *v*) as $\varOmega _{g,k}(u,v) = \mathcal {N}_k(p^{-1}\circ g^{-1} \circ p(u,v))$.

5 In the sense that $g(x+u,y+v) = g(x,y) + (u',v')$.

6 Better accuracy could be obtained by using image warping techniques. For example, sub-pixel accuracy can be obtained by up-sampling the permutation layer and then allowing the transformation filter to be translation variant (or, equivalently, by introducing a suitable non-linear mapping between the permutation layer and the transformation filters).

7 The Hellinger’s distance $(\sum _i (\sqrt{x_i}-\sqrt{y_i})^2)^{1/2}$ is preferred to the Euclidean distance as the HOG features are histograms.
